# Survival after severe amitriptyline poisoning with prolonged ventricular tachycardia and cardiac arrest

**DOI:** 10.1186/s13104-016-1963-0

**Published:** 2016-03-15

**Authors:** Dayakshi D. K. Abeyaratne, Chathuri Liyanapathirana, Anushka Gamage, Piyumanthi Karunarathne, Medhini Botheju, Jegarajah Indrakumar

**Affiliations:** Professorial Medical Unit, Colombo South Teaching Hospital, Kalubowila, Sri Lanka; Department of Medicine, Faculty of Medical Sciences, University of Sri Jayewardenepura, Nugegoda, Sri Lanka

**Keywords:** Tricyclic antidepressants, Cardiac arrhythmia, Prolonged cardiac arrest

## Abstract

**Background:**

Tricyclic antidepressants (TCA) are becoming one of the most frequently used substances in self poisoning. Significant morbidity and mortality associated with TCA overdose are often related to and refractory hypotension. We report the first case of survival after severe amitriptyline poisoning, leading to prolonged cardiac arrest and ventricular tachycardia (VT), resuscitated with 3 h of uninterrupted cardiac massage and Direct current (DC) shocks.

**Case presentation:**

A 25 year old girl presented with severe amitriptyline poisoning causing pulseless VT and prolonged cardiac arrest. After 3 h of uninterrupted external cardiac massage, together with nine DC shocks and intra venous bicarbonate injections the rhythm reverted to a nodal tachycardia, initial 2D echocardiogram showed left ventricular dysfunction, which recovered to normal after 2 weeks and the patient had a complete recovery subsequently.

**Conclusion:**

Our case highlights the importance of continued resuscitation in patients presenting with TCA poisoning and resistant arrhythmia, especially in young and otherwise healthy patients.

## Background

Tricyclic antidepressants (TCA) are used in management of a range of psychiatric disorders and is one of the most frequently abused drugs. It is often used as a sedative, especially in elderly population, commonly referred to as ‘sleeping tablets’. Therefore, its availability as an ‘over the counter’ drug, has lead to its’ higher usage in suicidal poisoning. Severe TCA overdose commonly causes fatal arrhythmia and myocardial depression due to blockade of sodium channels. This results in refractory hypotension, which is the commonest cause in death in TCA overdose [1, 2].

In literature, two cases were reported with severe imipramine poisoning with prolonged cardiac arrest managed with cardiac massage for 3 and 5 h [3, 4]. In the first case, cardiac arrest was due to prolonged asystole and in the second, resistant ventricular tachycardia (VT). The latter case is described in a 3-year-old, where they have failed to reverse VT with cardiac massage and has subsequently intervened with cardiac pacing to over-ride the arrhythmia.

We report the first case of severe amitriptyline poisoning with prolonged VT and cardiac arrest successfully reverted to sinus rhythm, with only continued external cardiac massage and repeated DC shocks for 3 h in a, low resource hospital system.

## Case history

**Fig. 1:**
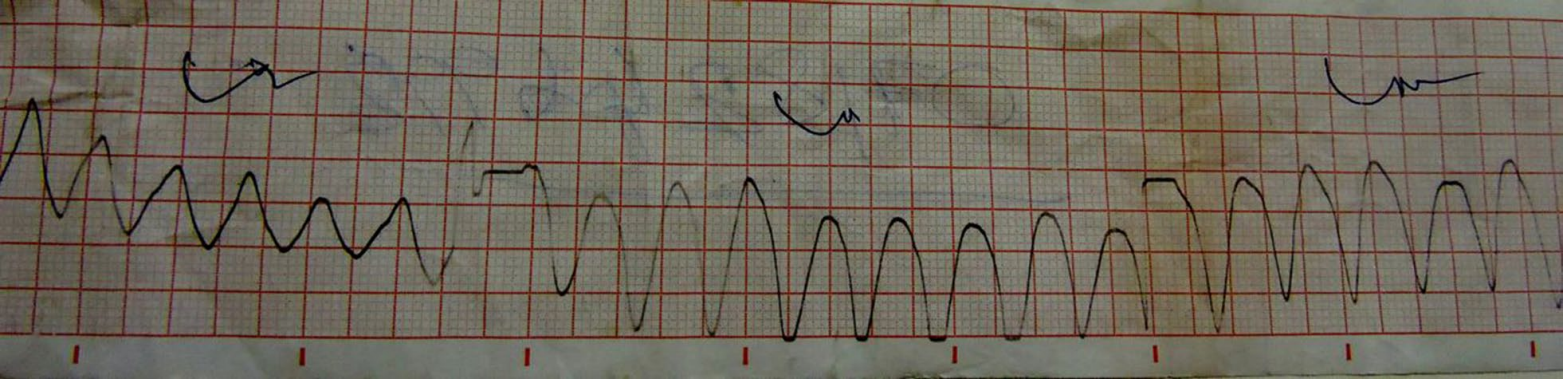
Two hours after poisoning—polymorphic VT

A 25 year old previously healthy girl without any co-morbidities, got admitted to the medical ward in an unconscious state after self-ingestion of an unknown quantity of “sleeping tablets”. Her Glasgow Coma Scale was 3/15, with dilated pupils. Her central and peripheral pulses were not felt and the blood pressure was un-recordable. Immediate cardiopulmonary resuscitation was started with external cardiac compressions and she was subsequently intubated and ventilated with 100 % oxygen. The cardiac monitor showed a VT with a rate varying between 150 and 180/min (Fig. 1). The history of “sleeping tablets” ingestion along with the clinical picture of dilated pupils and VT, immediately directed our suspicion towards TCA poisoning.

**Fig. 2:**
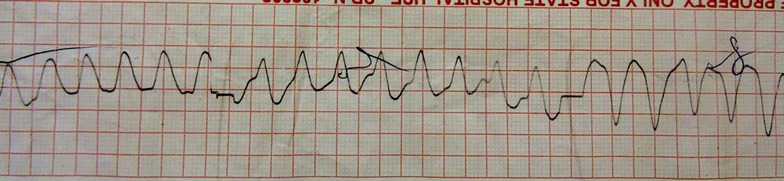
Four hours after poisoning with resuscitation—continuing VT

On detection of VT, she was given 150 joules, along with a bolus of 100 ml of intravenous sodium bicarbonate (8.4 %). Due to lack of response to initial treatment (Fig. 2), resuscitation was continued with cardiac massage for 3 h with repeated DC shocks up to nine times and IV sodium bicarbonate 50 ml boluses every 30 min. Gastric lavage was also performed concurrently. After 3 h the rhythm suddenly changed to nodal rhythm and the hemodynamic stability was achieved. At this stage the patient was transferred to intensive care unit where her mean arterial blood pressure was maintained above 65 mmHg with noradrenaline and dobutamine infusions. The sleeping tablets were subsequently identified as amitriptyline when her relatives discovered a pack of tablets (25 mg) from the place she was found unconscious and the quantity suspected was 20 tablets.

**Fig. 3:**
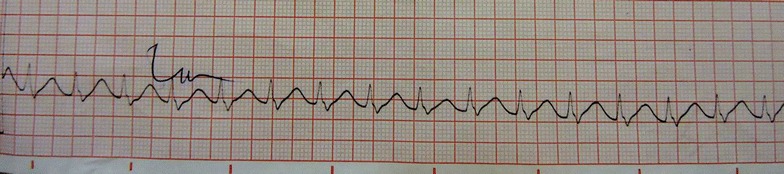
Ten hours after poisoning—prolonged QT with nodal tachycardia

**Fig. 4:**
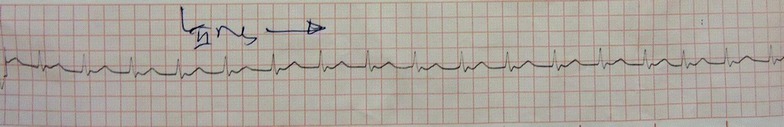
Thirty-six hours after poisoning—nodal tachycardia

**Fig. 5:**
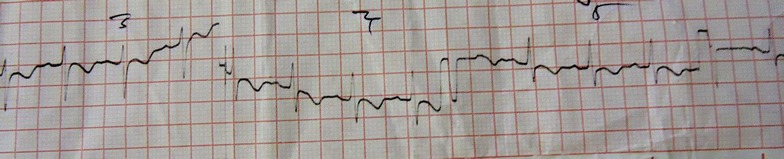
Day 4 after poisoning—nodal tachycardia with lateral T inversions

**Fig. 6:**
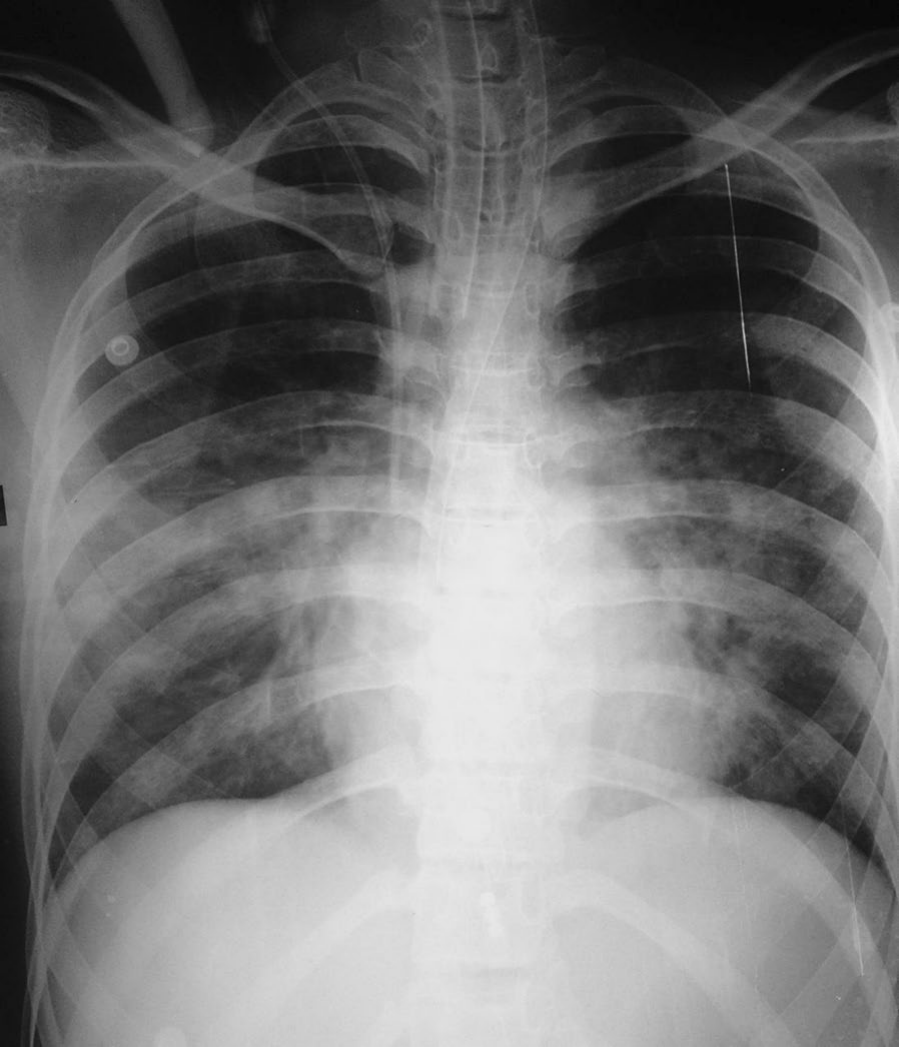
Twenty-four hour after poisoning—severe pulmonary edema

At the intesive care unit, she was ventilated for 48 h. Her blood pH was maintained in an alkaline state between 7.45 and 7.5 with intra-venous (IV) sodium bicarbonate injection. The post resuscitation nodal tachycardia (Figs. 3, 4) subsequently reverted to sinus rhythm along with T wave inversions in leads V_2_–V_6_ (Fig. 5). A 2D echocardiogram done at this stage showed an ejection fraction of 40 % with dilated left ventricle and global hypokinesia. Chest radiograph showed marked pulmonary edema (Fig. 6).

She was extubated on fourth day and was transferred to the medical ward for continuation of care. She was discharged after 1 week from the incident, when she obtained complete recovery. On questioning she could not recall any significant details prior to the incident, suggesting that she was having a retrograde amnesia of preceding 24 h from the incident. Her follow up ECGs done at 1 week and 1 month after discharge, showed sinus rhythm with normal T waves. The repeat 2D echocardiogram performed after 2 weeks showed improvement in her ejection fraction to 55 % with normal sized cardiac chambers. She did not have any other neurological deficits except for the short term retrograde amnesia that persisted.

## Discussion

TCAs remain a common cause of fatal drug poisoning over the years. They are rapidly absorbed from the gastrointestinal tract and are highly protein bound with a large volume of distribution. Therefore, it has a long half-life of elimination that generally exceeds 24 h [1]. The toxic effects of TCAs are caused by four main pharmacological mechanisms. These are: inhibition of norepinephrine (NE) reuptake at nerve terminals, direct alpha-adrenergic blockage, quinidine like effect on the myocardium and anticholinergic effect [1, 2].

In TCA poisoning, cardiovascular toxicity is evident by presence of arrhythmia and hypotension. It commonly causes prolongation of QT interval and Torsade de pointes, tachyarrhythmias or bradyarrhythmias. Tachyarrhythmias are commonly of ventricular origin such as VT or ventricular fibrillation or of supraventricular origin. Hypotension may be caused by reduced myocardial contractility and reduced systemic vascular resistance may be attributed to alpha-adrenergic blockade. Significant cardiac arrhythmia also contribute to hypotension and hemodynamic instability. In TCA overdose, ECGs changes that occur may predict the possibility of severe complications, even better than blood levels of TCA [5, 6]. However in our patient we were unable to check blood levels of amitriptyline, as the test is not readily available in the government sector.

Immediate treatment of cardiac arrhythmias involves correcting hypoxia, electrolyte abnormalities, hypotension and acidosis. But, intravenous sodium bicarbonate infusion is the mainstay of treatment in TCA poisoning, as it resolves cardiac arrhythmias, even in the absence of acidosis. The rationale for sodium bicarbonate therapy is based, in part upon animal studies showing, that it narrows the QRS complex, improves systolic blood pressure and controls ventricular arrhythmias [2]. In our patient intra venous sodium bicarbonate was given up to six boluses of 50 ml injections. Although severe TCA poisoning is associated with metabolic acidosis, secondary to myocardial dysfunction with hypotension, resulting in tissue hypo-perfusion and lactic acidosis, our patient did not have acidosis at any stage [4]. There are few cases reported, on severe TCA poisoning where failing initial resuscitation, they have tried new strategies such as intra venous lipid emulsion [7], percutaneous cardiopulmonary support [8] and use of intravenous magnesium sulfate [9]. But the actual benefits of these methods are yet to be proven.

The use of therapeutic hypothermia in the post resuscitation period had also been used in patients with severe TCA poisoning which had mainly been done to preserve the neurological functions in post- resuscitation period [10]. But in a low resource hospital setting, this is difficult until patient is admitted to an intensive care unit. There are cases reported on successful use of hypertonic saline in the management of severe TCA poisoning not responding to sodium bicarbonate, which is aimed at overwhelming the sodium channel blockade effect of TCA [11].

In our patient, continuous uninterrupted cardiac massage, successfully maintained cerebral perfusion during the arrest period. With the efforts of our highly enthusiastic resuscitation team, we were able to continue effective cardiac compressions for 3 h, even though it was thought that the recovery was a remote possibility as the time progressed. The prolonged hemodynamic instability may be attributable to slow metabolism and redistribution of the tricyclic during this time, with slow resolution of its effect on the myocardium.

We assume that the transient left ventricular dysfunction in our patient, was a result of tachycardia induced cardiomyopathy, due to persistent VT. This was evident in her, by the improvement of cardiac function to normal status once tachycardia was corrected. The direct effects of TCA too may have contributed to the cardiac failure [12]. Our patient recovered fully without complications except for the short term retrograde amnesia of 24 h preceding the incident. This retrograde amnesia has been previously reported in severe TCA poisoning with prolonged cardiac arrest [3].

Our case illustrates the importance of continuing external cardiac massage for many hours along with sodium bicarbonate therapy, in patients coming with severe TCA poisoning who are otherwise well and have a healthy myocardium. This is the first case report where prolonged resuscitation with cardiac compressions for 3 h and DC shocks with IV bicarbonate, saved a life without any cardiac interventions or other novel strategies.

## Conclusion

In severe TCA poisoning with prolonged cardiac arrest and VT, the continuity of primary resuscitation with uninterrupted cardiac massage with assisted ventilation as long as necessary, can be life saving. This case should serve as an example for current health care workers handling emergencies in a low resource setting, that prolonged resuscitation should be carried out with more enthusiasm, especially in young patients presenting with TCA poisoning.

## Consent

Written informed consent was obtained from the patient for publication of this case report and any accompanying images. A copy of the written consent is available for review.
